# Brucellosis As a Cause of Intestinal Perforation

**DOI:** 10.7759/cureus.7075

**Published:** 2020-02-22

**Authors:** Iffat Noureen, Muhammad Hamza, Huma Sabir Khan, Saleha Khan, Muhammad Hanif

**Affiliations:** 1 Surgical Unit II, Benazir Bhutto Hospital, Rawalpindi Medical University, Rawalpindi, PAK; 2 Surgery Unit II, Benazir Bhutto Hospital, Rawalpindi Medical University, Rawalpindi, PAK; 3 Surgical Unit II, Benazir Bhutto Hospital, Rawalpindi Medical Uninversity, Rawalpindi, PAK

**Keywords:** brucellosis, acute abdomen, intestinal perforation, appendicitis, ileostomy, ileitis

## Abstract

Brucellosis is a multisystem zoonotic infection. Patients usually presents with fever and nonspecific systemic symptoms but may rarely present with clinical presentation of an acute abdomen. In this case report, we present a 32-year-old male who presented to the emergency department with symptoms of acute abdomen. Exploration revealed ileal perforation secondary to brucellosis, for which loop ileostomy was fashioned.

## Introduction

Brucellosis is a zoonotic infection caused by facultative intracellular bacteria of the genus Brucella, which can involve multiple tissues and organs [1]. It is transmitted to humans from infected animals (cattle, sheep, goats, camels, pigs, or other animals) by ingestion of unpasteurized dairy products or by contact with infected fluids and tissues. It is a systemic infection with versatile presentations and systemic complications, among which gastrointestinal complications are very rare. This report presents a rare case of a young male patient who presented with peritonitis/acute abdomen caused by ileal perforation due to brucellosis.

## Case presentation

A 32-year-old, unmarried, nonsmoker male presented to the emergency department with generalized abdominal pain more marked in the right lower abdomen for one day. He had a history of high-grade fever associated with rigors and chills for 10 days. Fever was temporarily relieved by local antipyretics. He was otherwise fine with no history of nausea, vomiting, diarrhea, and constipation. He had no history of any urinary or genital symptoms. He had a history of consuming unpasteurized milk but had no history of animal contact. He was a shopkeeper by profession. He had no history of any medical illness, allergy, or surgery. There was no significant family or psychosocial history.

At presentation to the hospital, he had a pulse of 110 per minute, a blood pressure of 100/60 mmHg, a respiratory rate of 26 per minute, a temperature of 99°F, an oxygen saturation of 99% on room air, and random blood sugar of 105 mg/dl. The patient had an ill-look with pallor. The abdomen was generalized tender more in the right iliac fossa with guarding and rebound tenderness. Bowel sounds were absent. Psoas sign and obturator sign of acute appendicitis were positive. The rest of the systemic examination was unremarkable.

Lab investigations revealed that hemoglobin was 13 g/dl, total leukocyte count was 13,200 /mm^3^ (neutrophilia), and platelet count was 338,000 /mm^3^. Liver function tests, renal function tests, serum electrolytes, partial thromboplastin time (PTT), and activated PTT were normal. Hepatitis B and C profiles were negative. Ultrasound showed coarse liver with irregular margin, splenomegaly, and mild free fluid in the right iliac fossa with decreased intestinal peristaltic activity. Chest x-ray and abdominal x-ray had insignificant findings.

Based on this history, clinical examination, and investigation, diagnosis of acute appendicitis was established. The patient was prepared for open appendectomy in the emergency department of Rawalpindi Hospital, Pakistan. The patient and his attendants were counseled and informed consent was taken. The patient was explored using Gird-iron incision which was later extended to Rutherford incision. The operative finding was a single pinpoint perforation in ileum which was 12 cm from the ileocecal junction with minimal intra-abdominal contamination for which loop ileostomy was fashioned after washing the abdomen with adequate (5 l) normal saline. The rest of the bowel and viscera were normal. No drain was placed. The patient was admitted to the in-patient facility of the hospital.

During the patient stay in the hospital, the fever of the patient did not settle despite giving postoperative antibiotics like ceftriaxone 1 g IV BD, metronidazole 500 mg IV TDS, and azithromycin 500 mg IV BD. Urine R/E was insignificant, and urine culture was negative. The Typhidot IgM and IgG were negative. Malarial parasite thick and thin smears were negative.

After eight days of the first surgery, he developed fecal peritonitis for which re-exploration was done through a midline incision. A pinpoint perforation was seen in proximal ileum. There was extensive ileitis. A limited right hemicolectomy and ileocolostomy were done.

**Figure 1:**
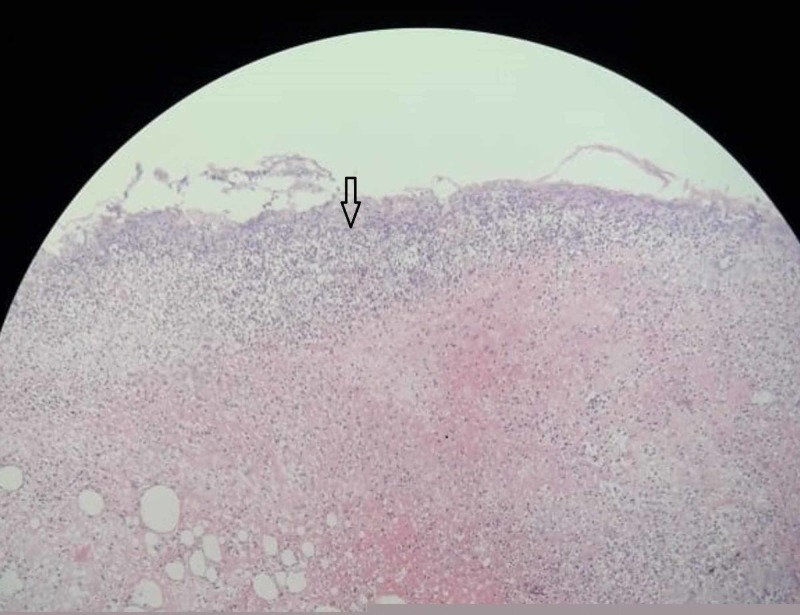
Photomicrograph (H&E, x10) shows section of perforation of ileum with neutrophilic infiltrates (pointer arrow) and areas of hemorrhage.

**Figure 2:**
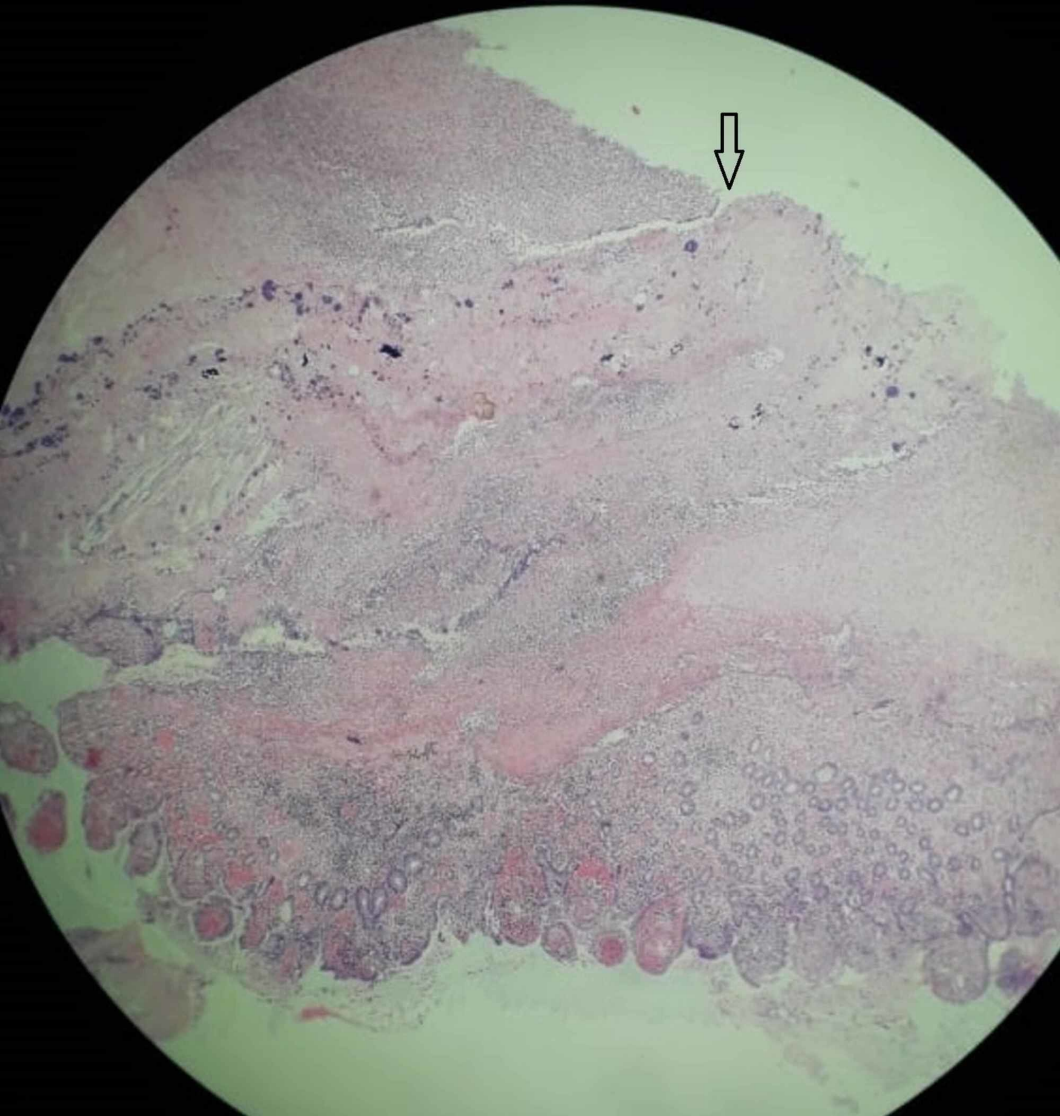
Photomicrograph (H&E, x40) shows segment of perforation of ileum with focal surface ulceration (arrow), suppuration, transmural inflammation, and congested blood vessels.

Despite the second exploration, his fever did not settle. Brucella IgG and IgM antibodies were measured by enzyme-linked immunosorbent assay (ELISA), which surprisingly came out to be reactive (positive). Brucella IgG was 2.43 (reference value was 0.9-1.1) and Brucella IgM was 1.89 (reference value was 0.9-1.1). Brucella antigen titer for Brucella abortus was 1:160 (positive) and Brucella antigen titre for Brucella mellitensis was 1:160 (positive). The blood culture report showed no growth. The patient was given tablet doxycycline 100 mg BD for six weeks, tablet rifampicin 600 mg OD for six weeks, tablet metronidazole 400 mg TDS for seven days, and tablet acetaminophen for fever. After taking these antibiotics, the patient started to improve. Histopathology report showed that the ileum section of perforation had surface ulceration, transmural inflammation, hemorrhages, suppuration, focal fibrosis, and congested blood vessels. The outer serosal surface had inflamed fibrinous exudate (Figures 1, 2).

The section of the terminal ileum had lymphoid hyperplasia of Peyer’s patch. The section of appendix had histopathological signs of acute appendicitis. The patient was seen at follow-up in the outpatient department with marked improvement in his general health.

## Discussion

Brucellosis, also known as ‘undulant fever’, is the most common zoonosis worldwide and an important public health problem in many developing countries [2]. Endemic areas for brucellosis include the Middle East, Central Asia, China, the Indian subcontinent, and Africa [3]. This bacterium is transmitted from animals to humans by ingestion of infected food products, direct contact with an animal, or inhalation of aerosols. Brucella species are aerobic gram-negative coccobacilli that possess a unique ability to invade both phagocytic and nonphagocytic cells and to survive in an intracellular environment by finding ways to avoid the immune system. This ability explains why brucellosis is a systemic disease and can involve almost every organ system [4].

Brucellosis may lead to a variety of clinical presentations. Fever and arthralgia of the large joints are the most common manifestations, followed by cough, malaise, myalgia, sweating, rash, and cardiac involvement. Being a systemic infection, it has multiple systemic manifestations. Although rare, gastrointestinal manifestations are diverse ranging from relatively milder complaints, such as anorexia, diarrhea, or constipation to more serious complications that have been reported in different case reports. It may present as an acute abdomen mimicking acute appendicitis, ileitis, infective colitis, acute cholecystitis, acute pancreatitis, mesenteric lymphadenitis, and intestinal obstruction [1,5-9].

Mesenteric lymphadenitis or inflammation and ulceration of Peyer's patches have been suggested as the possible mechanisms of Brucella-associated ileocolitis leading to perforation [1].

The clinical diagnosis of brucellosis can be difficult because of its nonspecific signs and symptoms so a high index of suspicion can lead to the correct diagnosis. It is important to get a detailed history which includes recent exposure to common host species of Brucella, especially cattle, sheep, goats, pigs, camels, buffaloes, or dogs; consumption of raw or inadequately cooked milk or milk products, meat, and offal derived from these animals. Occupational exposure, travel, or residence in an area in which the infection is prevalent also raises the probability of the diagnosis. The diagnosis is made with certainty when Brucella is recovered from the blood, bone marrow, or other tissues. Serological testing is the most commonly used method of diagnosing brucellosis among which the standard agglutination test is considered positive at titers of 1/160 or higher in case of active infection [5]. The Brucella ELISA is the most sensitive and specific serologic assay, and it may be positive when other tests are negative. Recently, polymerase chain reaction (PCR) has been developed for the detection of Brucella species in human blood specimens. A PCR test for samples other than blood has also been described [10]. Different tests can be joined to improve diagnostic yield [11]. A combination of PCR ELISA testing appears to be a highly sensitive and specific method for diagnosis [5].

The principle of the treatment of all forms of human brucellosis is the administration of effective antibiotics for an adequate length of time. The treatment option for the majority of brucellosis cases in adults and children (eight years of age and older) is doxycycline 100 mg twice a day for six weeks with streptomycin 1 g daily for two to three weeks or doxycycline 100 mg twice a day for six weeks with rifampicin 600-900 mg daily for six weeks [12,13].

## Conclusions

Brucellosis is an infection with multiple presentations. Therefore, clinical suspicion is required in cases presenting in endemic regions, especially when there is a history of exposure to animals and their products. This rare case report emphasizes that brucellosis is a preventable and treatable condition that must be considered in the differential diagnosis of acute abdomen and fever.
